# Astrocyte‐Derived Exosomal miR‐211‐5p Alleviates Blood–Brain Barrier Injury in a Rat Model of Traumatic Brain Injury

**DOI:** 10.1002/cns.70858

**Published:** 2026-04-09

**Authors:** Yan Chen, Xuan Yin, Erwei Zhang, Bin Li, Huijie Yu, Jiawang Qi, Minghao Liu, Meng Wang, Zhenzeng Fan, Lijun Yang

**Affiliations:** ^1^ Department of Neurosurgery The Second Hospital of Hebei Medical University Shijiazhuang Hebei China; ^2^ Department of Neurology The Eighth People's Hospital of Hebei Province Shijiazhuang Hebei China; ^3^ Department of Neurosurgery North China Oilfield General Hospital Renqiu China; ^4^ Department of Neurosurgery Tianjin Medical University General Hospital Tianjin China; ^5^ College of Forensic Medicine, Collaborative Innovation Center of Forensic Medical Molecular Identification, Hebei Key Laboratory of Forensic Medicine Hebei Medical University Shijiazhuang Hebei China

**Keywords:** astrocytes, brain trauma, exosomes, injury, neuroinflammation

## Abstract

**Background:**

Traumatic brain injury (TBI) triggers a cascade of secondary damage, including neuroinflammation, astrocyte activation, and disruption of the blood–brain barrier (BBB), all of which contribute to long‐term neurological deficits. Astrocyte‐derived exosomes have emerged as a promising therapeutic avenue; however, the specific contributions of their molecular cargo remain poorly understood. This study explores whether astrocyte‐derived exosomal delivery of microRNA‐211‐5p (miR‐211‐5p) can attenuate secondary injury and enhance functional recovery following TBI.

**Methods:**

Primary astrocytes were transfected with AAV‐rno‐miR‐211‐5p, and the resulting exosomes were isolated and characterized. TBI was induced in adult rats using a controlled cortical impact (CCI) model. Exosomes (1 × 10^11^ particles) were administered intravenously 30 min post‐injury. Behavioral assessments were conducted to evaluate cognitive function and neurological deficits. Brain edema, glial activation, and the expression of inflammatory cytokines (IL‐6, IL‐1β, TNF‐α) and BBB‐related markers—including glial fibrillary acidic protein (GFAP), matrix metalloproteinase 9 (MMP9), aquaporin 4 (AQP4), and the tight junction proteins zonula occludens‐1 (ZO‐1) and claudin‐5—were analyzed using quantitative real‐time PCR, Western blotting, enzyme‐linked immunosorbent assay, and histopathological techniques.

**Results:**

Exosomes enriched with miR‐211‐5p significantly improved cognitive and neurological outcomes, reduced cerebral edema, and downregulated the expression of GFAP, MMP9, and AQP4. Furthermore, the integrity of the BBB was preserved, as evidenced by sustained expression of ZO‐1 and claudin‐5. Levels of the proinflammatory cytokines IL‐6, IL‐1β, and TNF‐α were also markedly decreased in the injured cortex.

**Conclusion:**

Astrocyte‐derived exosomal miR‐211‐5p confers neuroprotection in TBI by modulating glial activation, reducing neuroinflammation, and preserving BBB integrity. These findings underscore the therapeutic potential of miR‐211‐5p‐loaded exosomes as a cell‐free, targeted intervention for brain trauma.

## Graphical abstract

Astrocyte‐derived exosomes enriched with miR‐211‐5p significantly improved neurological and cognitive outcomes after traumatic brain injury in rats. Treatment reduced astrocyte activation, neuroinflammation, brain edema, and MMP9/AQP4 expression while preserving blood–brain barrier integrity. These findings highlight miR‐211‐5p–loaded exosomes as a promising cell‐free therapy for TBI.

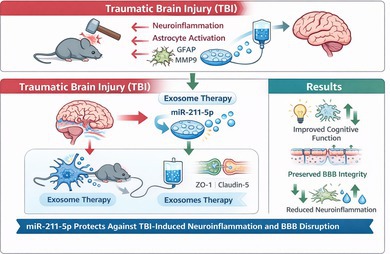

## 1 Introduction

Traumatic brain injury (TBI) is a leading cause of mortality and long‐term disability, particularly among young adults and the elderly [1, 2]. Besides the immediate mechanical insult, TBI initiates a cascade of secondary injury processes, including neuroinflammation, oxidative stress, cerebral edema, and blood–brain barrier (BBB) disruption, which collectively contribute to progressive neurological decline [3]. Despite decades of research, therapeutic options for TBI remain limited, and current clinical interventions are largely supportive rather than curative [2]. A major obstacle in the development of effective treatments is the lack of targeted strategies that address the complex cellular and molecular interactions in the injured brain [4].

Astrocytes, the most abundant glial cell type in the central nervous system, play a pivotal role in modulating the post‐injury environment [5]. Upon TBI, astrocytes become reactive, a process characterized by morphological changes and upregulation of glial fibrillary acidic protein (GFAP) [6]. While reactive astrocytes can initially serve protective functions, excessive or prolonged activation contributes to glial scar formation [7], neuroinflammation, and impaired neural regeneration [5]. Although several studies have investigated astrocytic responses following brain injury, the mechanisms by which astrocytes communicate with surrounding cells and influence neuroinflammatory and regenerative processes remain incompletely understood [8].

Exosomes—small extracellular vesicles ranging from 30 to 150 nm—have recently gained attention as potent mediators of intercellular communication [9]. Astrocyte‐derived exosomes carry diverse cargos, including proteins, lipids, and microRNAs (miRNAs), and can influence neuronal survival, glial activation, and immune responses [10]. However, previous studies exploring the therapeutic potential of exosomes in TBI models have often treated them as homogenous entities without characterizing or modulating their molecular content, hampering their translational value. Furthermore, many investigations fail to distinguish whether observed therapeutic effects are driven by exosome structure, general cargo, or specific signaling molecules.

Among regulatory miRNAs, miR‐211‐5p has recently emerged as a candidate modulator of inflammation and oxidative injury in various neurological conditions [11]. Yet, its functional role in astrocyte‐derived exosomes after TBI has not been elucidated [12]. Most existing studies on miR‐211‐5p have focused on tumor biology [13] or ischemic models [14, 15], leaving a gap in understanding its relevance to traumatic injuries and glial signaling. Additionally, no studies to date have systematically investigated the delivery of miR‐211‐5p via astrocyte‐derived exosomes and its influence on BBB integrity, glial activation, and post‐injury cognitive outcomes in vivo.

To address these gaps, we investigated the therapeutic potential of exosomes released from primary astrocytes overexpressing miR‐211‐5p in a rat model of TBI. We hypothesized that targeted enrichment of miR‐211‐5p in astrocyte‐derived exosomes could modulate secondary injury mechanisms and promote functional recovery. This study provides a mechanistic framework for understanding the role of exosomal miR‐211‐5p in TBI and highlights its promise as a novel therapeutic approach for mitigating the detrimental consequences of brain trauma.

## 2 Methods

### 2.1 Isolation and Cultivation of Primary Astrocytes

Animal studies were approved by the Research Ethics Committee of the Second Hospital of Hebei Medical University (#2022‐R258). Cortical tissues were collected from postnatal day 1–3 Sprague–Dawley rats and enzymatically dissociated in DMEM containing 0.25% trypsin at 37°C for 30 min. The cell suspension was centrifuged at 200 × g for 5 min, and the resulting pellet was resuspended in DMEM supplemented with 10% fetal bovine serum and antibiotics. Cells were plated onto poly‐D‐lysine‐coated flasks and cultured at 37°C in a humidified atmosphere with 5% CO_2_. After reaching confluency, cultures were shaken to remove non‐astrocytic cells. Immunostaining with anti‐GFAP antibody was used to verify astrocyte identity.

### 2.2 miR‐211‐5p Overexpression In Astrocytes

To increase miR‐211‐5p levels in astrocytes, cells were transduced with an adeno‐associated viral (AAV) vector encoding rno‐miR‐211‐5p (AAV‐rno‐miR‐211‐5p) purchased from Shanghai Jikai Gene Chemical Technology Co. Ltd. The viral vector was directly applied to cultured astrocytes according to the manufacturer's protocol. Transduction efficiency was confirmed by RT‐qPCR 72 h post‐transduction.

### 2.3 miR‐211‐5p Inhibition In Vivo

To verify the specificity of miR‐211‐5p‐mediated protective effects, an AAV‐rno‐miR‐211‐5p‐inhibition virus (miR‐sponge) was purchased from Shanghai Jikai Gene Chemical Technology Co. Ltd. The viral vector was AAV2/9 with a glial cell‐specific promoter GfaABC1D and a titer of 1 × 10^12^ vg/mL. The miR‐sponge mechanism involves constitutive expression of sequences complementary to miR‐211‐5p, competitively sequestering miR‐211‐5p and specifically blocking its biological activity. The virus was administered via stereotactic injection simultaneously with EXO^miR^ intravenous injection, i.e., within 30 min after TBI modeling, ensuring that miR‐211‐5p could be effectively inhibited when EXO^miR^ exerted its effects. Using the stereotactic injection technique, the virus solution was slowly injected at 0.2 μL/min via a microinjector. After injection completion, the needle remained in place for 5 min to prevent virus backflow. Each rat received 1 × 10^11^ vg of the virus (5 μL volume) in the unilateral injury region.

### 2.4 Exosome Collection and Analysis

Exosomes were harvested by culturing astrocytes in serum‐free or exosome‐depleted media for 72 h. The conditioned medium was cleared by sequential centrifugation steps to remove cells and debris, followed by ultracentrifugation at 100,000 g to isolate exosomal vesicles. Pellets were washed with PBS and stored at −80°C. Vesicle size was determined by nanoparticle tracking analysis. Western blotting confirmed the expression of exosomal markers, including Alix, TSG101, and CD63.

### 2.5 Traumatic Brain Injury Model and Treatment Protocol

Male Sprague–Dawley rats (250–280 g) were used to establish a moderate TBI model. A controlled cortical impact was performed under isoflurane anesthesia. Thirty minutes post‐injury, exosomes (1 × 10^11^ particles in 300 μL PBS) or PBS alone were injected into the right jugular vein, followed by a PBS flush.

### 2.6 Evans Blue Permeability Assay

BBB integrity was evaluated using the Evans blue extravasation assay with three biological replicates per group. On post‐injury day 2, 2% Evans blue solution in PBS (2 mL/kg) was injected via the rat tail vein. Twenty‐four hours after injection (post‐injury day 3), rats were anesthetized and transcardially perfused with saline until the outflow from the right atrium was clear. The ipsilateral cortex was then dissected, weighed, and homogenized. The absorbance of the homogenate was measured at 620 nm using a spectrophotometer, and Evans blue concentration was calculated based on a standard curve and expressed as μg/g of tissue weight. Gross brain specimens were photographed to document dye distribution.

### 2.7 Behavioral Assessment

All behavioral assessments were conducted using a double‐blind design to minimize subjective bias and ensure objectivity and reliability of the results. Animal grouping was performed by an independent experimenter using a random number table, with group information recorded only as random codes without any group identifiers. Personnel performing behavioral tests and data analysts did not participate in prior animal modeling, exosome administration, or grouping, and were only aware of animal codes, not treatment groups. During testing, operators followed unified standard procedures, with data collection performed via automated software for initial recording. Subsequent statistical analysis was conducted by another independent person, with group code decryption and result matching performed only after all data processing was complete. Cognitive function was tested using the Morris Water Maze starting two weeks post‐injury. Rats completed four trials daily for five days to assess escape latency and swimming ability. On day six, a probe test was conducted to evaluate memory retention by measuring the time spent in the target quadrant and the number of platform crossings.

### 2.8 Brain Water Content Measurement

Brain edema was quantified 3 days post‐injury using the wet/dry method. Tissue sections from the injured cortex were weighed immediately (wet weight), dried at 100°C for 24 h, and reweighed (dry weight). Water content was calculated as:

$$\left(\mathrm{wet}\;\text{weight}-\mathrm{dry}\;\text{weight}\right)/\mathrm{wet}\;\text{weight}\times 100\%.$$

### 2.9 Neurological Deficit Assessment

Neurological function was evaluated using the modified Neurological Severity Score (mNSS), which comprehensively assesses four core domains: Motor function, sensory function, reflexes, and balance ability. The total score ranges from 0 to 18, with higher scores indicating more severe neurological deficits. Assessments were conducted at baseline (before TBI) and at 3, 7, and 14 days post‐injury. All evaluations were performed by blinded investigators who did not participate in experimental grouping to avoid subjective bias. Motor function assessment included spontaneous activity, limb muscle strength (forelimb grasping, hindlimb support), and walking posture. Sensory function was tested for pain response (tail pinch) and tactile response (limb touch). Reflex evaluation included corneal reflex, pupillary light reflex, and righting reflex. Balance ability was assessed by observing standing stability and walking trajectory on a horizontal smooth surface.

### 2.10 Protein and Cytokine Quantification

Cortical tissues were homogenized in PBS containing protease inhibitors and centrifuged to remove debris. Supernatants were stored at −80°C. *ELISA* kits were used to quantify the levels of IL‐6, IL‐1β, and TNF‐α. For protein expression analysis, Western blotting was performed using antibodies against GFAP, MMP9, AQP4, ZO‐1, Claudin‐5, and GAPDH.

### 2.11 Quantitative PCR

Total RNA was isolated using TRIzol, and cDNA was synthesized for qPCR. The following primers were used: miR‐211‐5p (CGTTCCCTTTGTCATCCTTGCCT), U6 (Forward: CCTGCTTCGGCAGCACA; Reverse: AACGCTTCACGAATTTGCGT), GFAP (Forward: AGAAGCTCCAAGATGAACCG; Reverse: TCCTTGAGGTTCTCTTGCTG), AQP4 (Forward: CCAGCTGTGATTCCAAAACGGAC; Reverse: TCTAGTCATACTGAAGACAATACCTC), MMP9 (Forward: CCTGGAGACCTGAGAACCA; Reverse: GGCTGTCCAGTCTCAGCAT), GAPDH (Forward: TGCCCACTACGACAATTTCC; Reverse: CCTGTTGCTGTAGCCAAATT). Gene expression levels were normalized to U6 or GAPDH using the 2^−ΔΔCt^ method.

### 2.12 Statistical Analysis

All quantitative data are presented as mean ± standard deviation (SD). Group comparisons were performed using an unpaired two‐tailed Student's *t*‐test with Welch's correction for two‐group comparisons or Brown–Forsythe one‐way ANOVA followed by Dunnett's T3 multiple comparisons test for comparisons among more than two groups. Behavioral data from the Morris Water Maze were analyzed using repeated‐measures ANOVA. A *p*‐value of less than 0.05 was considered statistically significant. Statistical analyses and graph generation were conducted using GraphPad Prism (version 9.0).

## 3 Results

### 3.1 Overexpression of miR‐211‐5p in Astrocytes and Astrocyte‐Derived Exosomes

**FIGURE 1:**
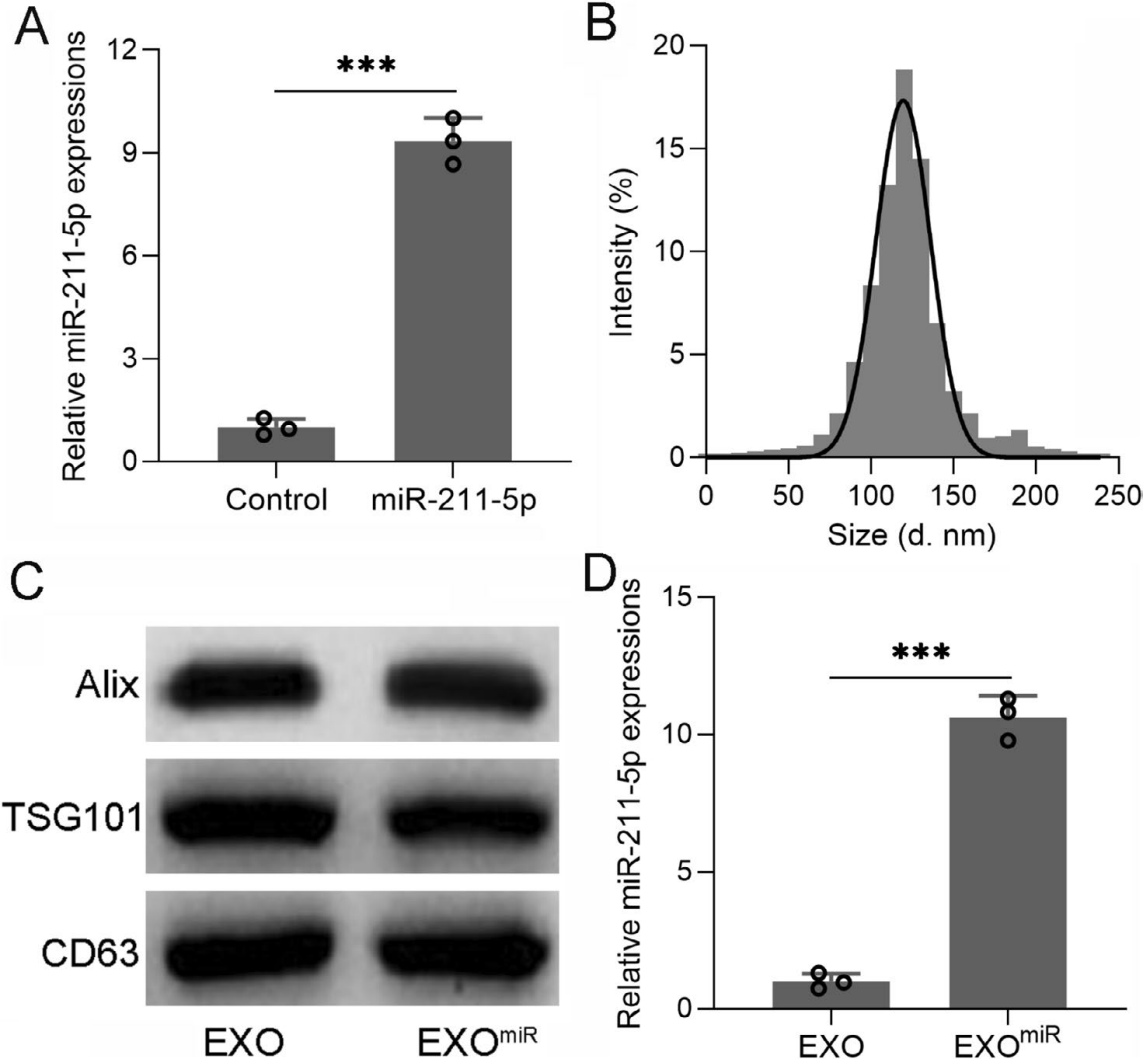
Overexpression of miR‐211‐5p in primary astrocytes and astrocyte‐derived exosomes. (A) RT‐qPCR was used to analyze the expression of miR‐211‐5p from primary astrocytes (control) or astrocytes transfected with AAV‐rno‐miR‐211‐5p for 48 h. (B) Size of the particles in the isolated exosome mixture obtained through nanoparticle tracking analysis. (C) Western blotting was used to measure the protein expressions of Alix, TSG101, and CD63 from exosomes isolated from primary astrocytes or astrocytes transfected with AAV‐rno‐miR‐211‐5p for 48 h. (D) RT‐qPCR was used to analyze the expression of miR‐211‐5p in exosomes isolated from primary astrocytes or astrocytes transfected with AAV‐rno‐miR‐211‐5p for 48 h. Data were shown with mean ± SD. ***p < 0.001 vs. control or EXO group from Unpaired *t*‐test with Welch's correction.

Transfection with AAV‐rno‐miR‐211‐5p significantly increased miR‐211‐5p levels in primary astrocytes as determined by RT‐qPCR (Figure 1A, *p <* 0.001). Nanoparticle tracking analysis indicated that the size distribution of these vesicles was consistent with exosomes, predominantly ranging from 100 to150 nm (Figure 1B). Western blot analysis showed positive expression of canonical exosomal markers Alix, TSG101, and CD63 in both control and miR‐211‐5p‐overexpressing exosome groups (Figure 1C). Notably, exosomes derived from astrocytes transfected with AAV‐miR‐211‐5p showed a significant enrichment of miR‐211‐5p compared to control exosomes (Figure 1D, *p <* 0.001).

### 3.2 Astrocyte‐Derived Exosomal miR‐211‐5p Ameliorates Cognitive Deficits in TBI Rats

**FIGURE 2:**
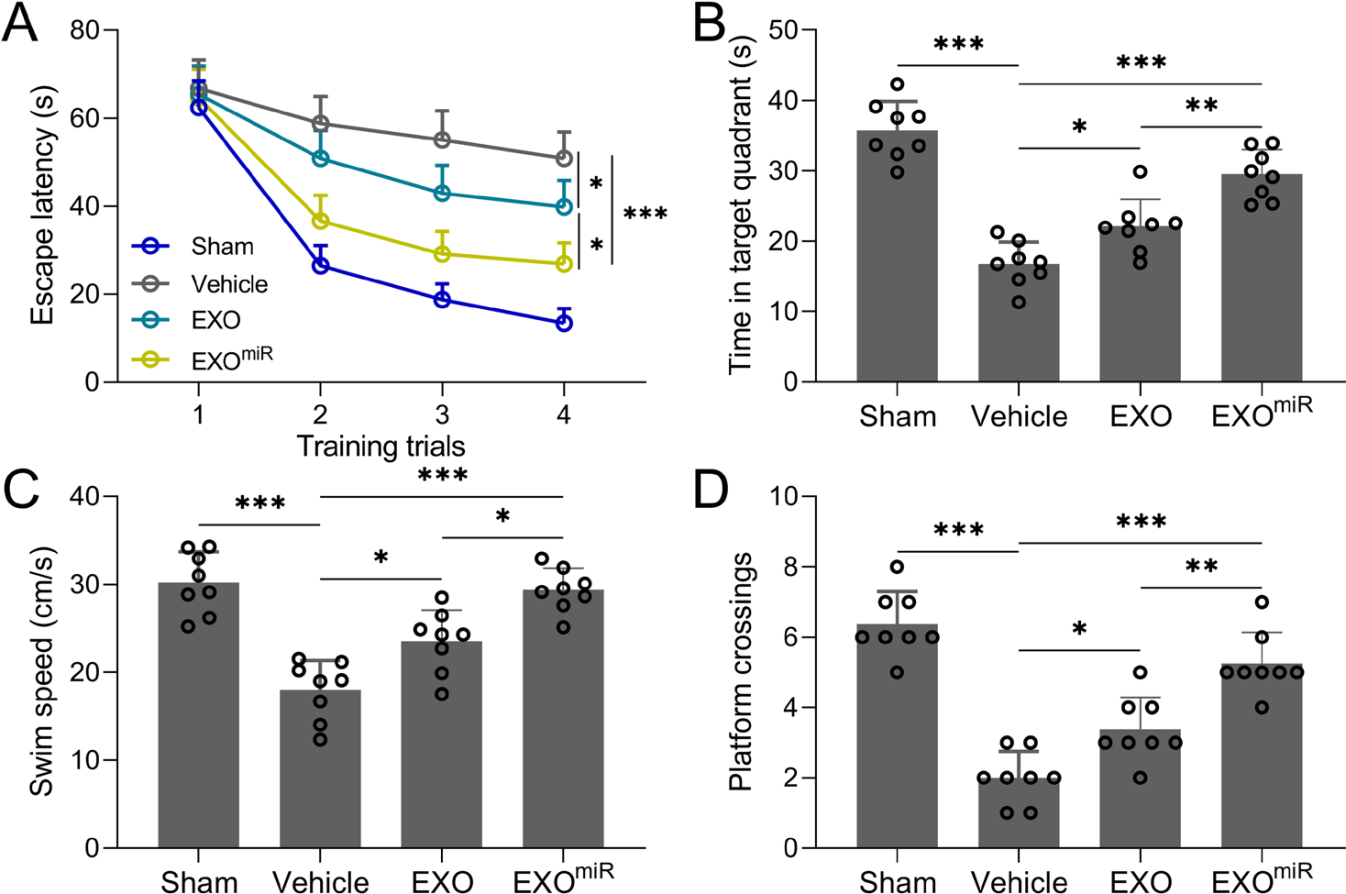
Astrocyte‐derived exosomal miR‐211‐5p protected against cognitive impairments in TBI rats. In 4 trails of the Morris water maze test, the rats' escape latencies (A) and the average swim speed (C) were measured. In the probe trial, the time in the target quadrant in 60 s (B) and the number of platform site crossings (D) were recorded. *N* = 8 for each group. Data were shown with mean ± SD. *p < 0.05, **p < 0.01, and ***p < 0.001 from Brown–Forsythe ANOVA test followed by Dunnett's T3 multiple comparisons test.

To assess the functional impact of astrocyte‐derived exosomal miR‐211‐5p in vivo, Morris Water Maze (MWM) tests were performed 14 days post‐TBI. Rats treated with exosomes overexpressing miR‐211‐5p exhibited significantly reduced escape latencies across the training trials compared to control TBI rats (Figure 2A, *p <* 0.001). Probe trial analysis revealed that exosomal miR‐211‐5p treatment significantly increased both time spent in the target quadrant (Figure 2B, *p <* 0.001) and the number of platform site crossings (Figure 2D, *p <* 0.001) when compared to control TBI rats, indicating improved spatial memory. Although swimming speed showed inter‐group differences (Figure 2C, *p <* 0.001 for the comparison between EXO^miR^ and vehicle groups), these differences do not confound the interpretation of cognitive outcomes. Swimming speed primarily reflects motor ability, physical state, and motivation to escape the water environment, serving as a non‐cognitive auxiliary indicator. The reduced swimming speed in the vehicle group was directly related to impaired motor‐related neural networks and decreased limb coordination after trauma. These findings suggest that miR‐211‐5p‐enriched exosomes confer stronger neuroprotective effects than unmodified astrocyte‐derived exosomes.

### 3.3 Exosomal miR‐211‐5p Improves Neurological Function and Reduces Brain Edema

**FIGURE 3:**
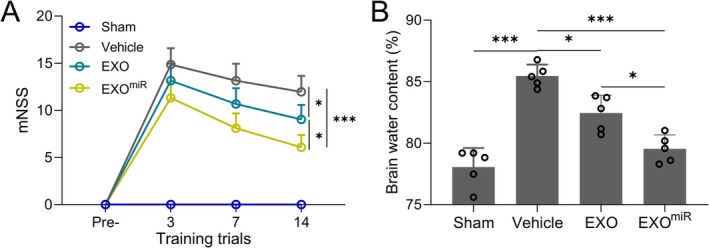
Astrocyte‐derived exosomal miR‐211‐5p protected TBI‐induced neurological deficit (A) and brain edema (B) in rats. Brain water content was compared 3 days post‐TBI, and neurological deficit scores were measured pre, 3, 7, and 14 days post‐TBI. Data are presented as mean ± SD. *N* = 8 in A and 5 in B. Data were shown with mean ± SD. *p < 0.05 and ***p < 0.001 from Brown–Forsythe ANOVA test followed by Dunnett's T3 multiple comparisons test.

Assessment of neurological deficits using a standardized scoring scale revealed a significant improvement in functional outcomes in TBI rats receiving miR‐211‐5p‐enriched exosomes at days 3, 7, and 14 post‐TBI compared to vehicle‐treated TBI rats (Figure 3A, *p <* 0.001). Additionally, brain water content measured at day 3 post‐injury showed a marked reduction in edema in the EXO^miR^ group relative to the vehicle group (Figure 3B, *p <* 0.001), indicating that the treatment mitigated brain swelling.

### 3.4 Accumulation of ExomiR in Injured Cortex

The in vivo biodistribution of EXO^miR^ was confirmed through near‐infrared fluorescence imaging (Figure S1), demonstrating brain‐targeted enrichment of systemically administered exosomes in the injured cortex. DiR‐labeled EXO^miR^ exhibited persistent fluorescence signals in the brain region up to 7 days post‐injection, supporting the notion that intravenously delivered astrocyte‐derived exosomes can cross the compromised BBB and accumulate at injury sites. This brain‐targeting property, combined with the observed regulation of BBB‐related molecules (MMP9/AQP4, tight junction proteins), provides strong evidence that EXO^miR^ exerts therapeutic effects directly within the injured brain parenchyma. While specific cellular uptake patterns remain to be fully characterized, the functional outcomes and molecular changes observed in this study are consistent with effective delivery and bioactivity of exosomal miR‐211‐5p in the TBI microenvironment.

### 3.5 Inhibition of Astrocyte Activation by Exosomal miR‐211‐5p

**FIGURE 4:**
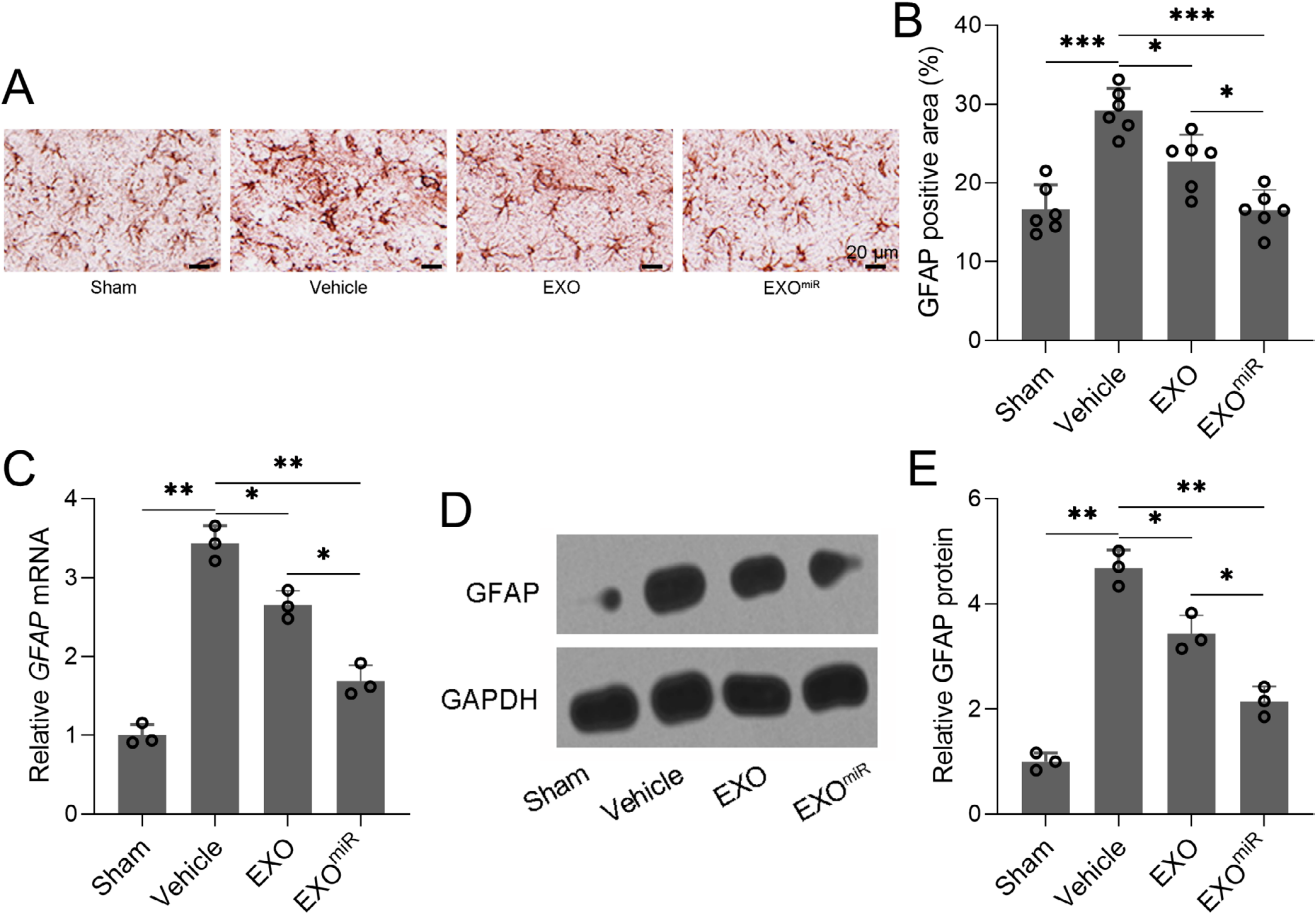
Astrocyte‐derived exosomal miR‐211‐5p protected against traumatic brain injury induced astrocyte activation in the ipsilateral cortex of experimental rats. (A) Representative immunohistochemical analyses of astrocytes (GFAP) in ipsilateral cortex sections 3 days post‐TBI, and (B) GFAP‐positive cells area in ipsilateral cortex sections 3 days post‐TBI. *N* = 6 for each group. (C) The mRNA expressions of GFAP in the ipsilateral cortex were measured by qRT‐PCR. (D) Western blotting was used to measure the protein expression of GFAP in the ipsilateral cortex. GAPDH was used as a loading control, and the expressions were normalized to Sham (E). 8 rats in each group were used, and the tissues were homogenized for 3 repeated experiments. Data were shown with mean ± SD. *p < 0.05, **p < 0.01, and ***p < 0.001 from Brown–Forsythe ANOVA test followed by Dunnett's T3 multiple comparisons test.

Three days after TBI, GFAP immunohistochemistry revealed elevated astrocyte activation in the ipsilateral cortex of TBI rats, which was attenuated by administration of exosomal miR‐211‐5p (Figure 4A,B, *p <* 0.001). This was further supported by significant reductions in both GFAP mRNA (Figure 4C, *p* = 0.006) and protein (Figure 4D,E, *p =* 0.008) levels in the cortex of exosomal miR‐211‐5p‐treated rats, suggesting that exosomal delivery of miR‐211‐5p effectively suppresses reactive astrocytosis following TBI.

### 3.6 Exosomal miR‐211‐5p Preserves Blood–Brain Barrier Integrity by Targeting MMP9 and AQP4

Evans blue extravasation assay demonstrated significant BBB protection by EXO^miR^ treatment (Figure S2). Gross imaging of brain tissues showed marked blue staining in the Vehicle group, which was significantly reduced in the EXO group and further attenuated in the EXO^miR^ group. Quantitative analysis revealed that Evans blue concentration in the ipsilateral cortex was significantly lower in the EXO^miR^ group compared to the vehicle group, indicating preserved BBB integrity. Administration of miR‐sponge (EXO^miR^ + miR‐sponge group) significantly increased Evans blue concentration compared to the EXO^miR^ group, demonstrating that the BBB protective effect is specifically dependent on miR‐211‐5p.

**FIGURE 5:**
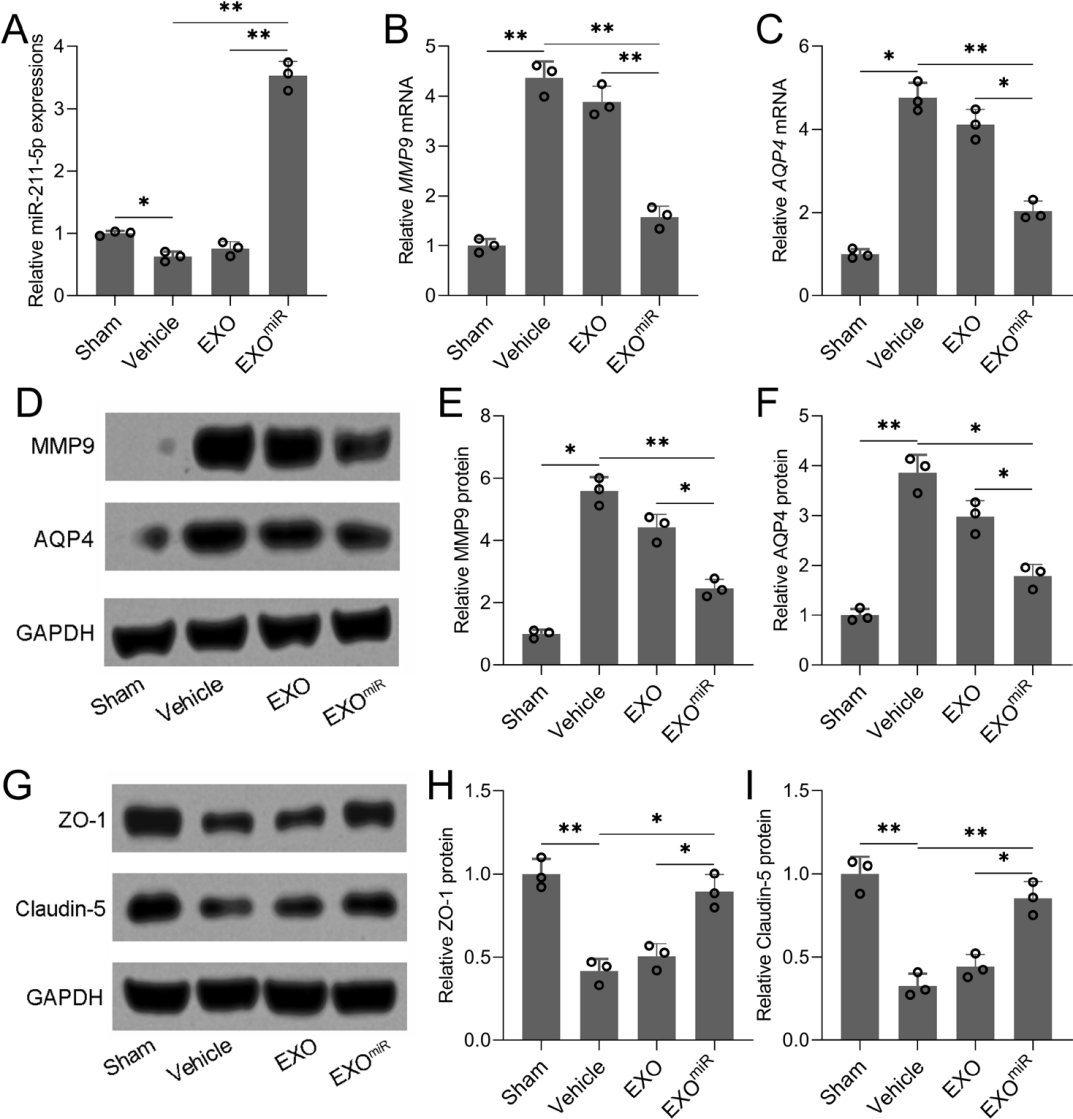
Astrocyte‐derived exosomal miR‐211‐5p inhibited MMP9/AQP4 and protected BBB integrity in TBI rats. RT‐qPCR was used to analyze the expression of miR‐211‐5p (A), mRNA expressions of MMP9 (B) and AQP4 (C) in the ipsilateral cortex. (D and G) Western blotting was used to measure the protein expressions of MMP9, AQP4, ZO‐1, and Claudin‐5 in the ipsilateral cortex. GAPDH was used as a loading control, and the expressions were normalized to Sham (E, F, H, I). 8 rats in each group were used, and the tissues were homogenized for 3 repeated experiments. Data were shown with mean ± SD. *p < 0.05, **p < 0.01 from Brown–Forsythe ANOVA test followed by Dunnett's T3 multiple comparisons test.

In the ipsilateral cortex, miR‐211‐5p expression was significantly increased in the EXO^miR^ group compared to the vehicle group (Figure 5A, *p =* 0.007). Correspondingly, both mRNA and protein levels of matrix metalloproteinase 9 (MMP9) and aquaporin 4 (AQP4) were markedly reduced in the EXO^miR^ group when compared to the vehicle group (Figure 5B–F; *p* = 0.009 and *p* = 0.005 for MMP9 and AQP4 mRNA comparison, respectively; *p* = 0.003 and *p* = 0.017 for MMP9 and AQP4 protein comparison, respectively). Furthermore, miR‐sponge treatment (EXO^miR^ + sponge group) abolished the suppressive effects of EXO^miR^ on MMP9 and AQP4 expression (Figure S3B–C). Western blotting further demonstrated that the expression of tight junction proteins ZO‐1 and Claudin‐5 was upregulated in the EXO^miR^ group (Figure 5G–I, *p =* 0.021 and *p* = 0.005, respectively). Notably, astrocyte‐derived exosomes without miR‐211‐5p overexpression did not significantly affect MMP9/AQP4 expression or tight junction protein levels, suggesting that the observed protection of BBB integrity is specifically mediated by miR‐211‐5p.

### 3.7 Exosomal miR‐211‐5p Suppresses Neuroinflammation Post‐TBI

**FIGURE 6:**
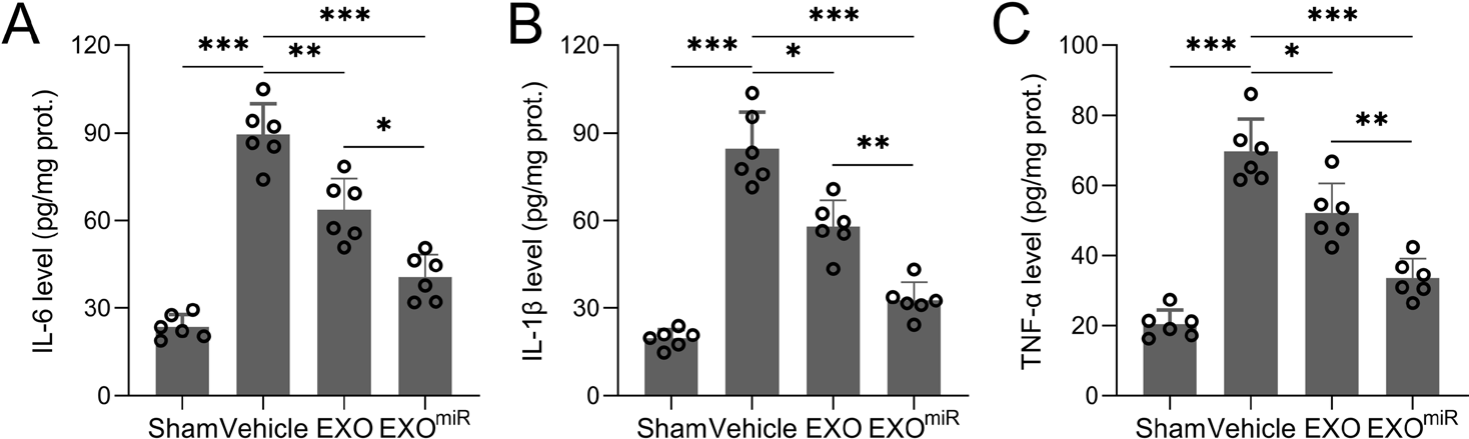
Astrocyte‐derived exosomal miR‐211‐5p inhibited inflammation in TBI rats. Protein levels of IL‐6 (A), IL‐1β (B), and TNF‐α (C) in the ipsilateral cortex were measured by ELISA. *N* = 6 for each group. Data were shown with mean ± SD. *p < 0.05, **p < 0.01, and ***p < 0.001 from Brown–Forsythe ANOVA test followed by Dunnett's T3 multiple comparisons test.

To investigate the anti‐inflammatory potential of exosomal miR‐211‐5p, levels of inflammatory cytokines in the ipsilateral cortex were quantified by ELISA. Treatment with miR‐211‐5p‐enriched exosomes significantly reduced the expression of IL‐6 (Figure 6A, *p <* 0.001), IL‐1β (Figure 6B, *p <* 0.001), and TNF‐α (Figure 6C, *p <* 0.001) in the TBI rats, indicating a pronounced attenuation of TBI‐induced neuroinflammation. While astrocyte‐derived exosomes alone provided partial benefit, the anti‐inflammatory effects were significantly greater in the miR‐211‐5p‐enriched group.

## 4 Discussion

This study demonstrates that astrocyte‐derived exosomes enriched with miR‐211‐5p confer significant neuroprotective effects in TBI. Specifically, miR‐211‐5p overexpression in primary astrocytes led to the successful packaging and secretion of this regulatory miRNA into exosomes, which, when administered intravenously after injury, mitigated multiple hallmarks of secondary brain damage. These included reduced cognitive deficits, decreased brain edema, suppression of GFAP upregulation, improved neurological scores, preservation of BBB integrity, and dampening of pro‐inflammatory cytokine expression in the injured cortex.

While numerous studies have examined the role of astrocyte‐derived exosomes in modulating post‐injury responses [5, 11, 16, 17], many have relied on unmodified exosome preparations without interrogating or manipulating their cargo. This limits mechanistic insight and reproducibility across experimental models. In contrast, our study provides direct evidence that targeted enrichment of miR‐211‐5p in astrocyte‐derived exosomes is responsible for their therapeutic efficacy. Previous research has associated miR‐211‐5p with anti‐inflammatory and anti‐apoptotic roles in ischemic injury and cancer models [18, 19], yet its role in the context of TBI and glial signaling has remained unexplored. Our findings thus expand the scope of miR‐211‐5p as a bioactive molecule with potential relevance in acute CNS trauma. Our study demonstrates that targeted enrichment of miR‐211‐5p enhances the therapeutic efficacy of astrocyte‐derived exosomes. The direct targeting relationship between miR‐211‐5p and MMP9 was previously validated in our published work through luciferase reporter assays, which confirmed that miR‐211‐5p directly binds to the 3′ UTR of MMP9 and inhibits its expression [12]. The current study extends these findings to in vivo validation in TBI models, demonstrating that exosomal delivery of miR‐211‐5p effectively reduces MMP9 and AQP4 expression, with this effect being abolished by miR‐sponge treatment (Figure S3), further confirming the specificity of this regulatory axis.

Compared to prior studies that reported modest improvements in TBI outcomes using unmodified glial exosomes, the enhanced suppression of MMP9 and AQP4 expression, coupled with upregulation of ZO‐1 and Claudin‐5, underscores the specific capacity of miR‐211‐5p to preserve BBB structure and function [12, 20]. Notably, the absence of significant effects from naïve astrocyte‐derived exosomes on these markers further strengthens the conclusion that miR‐211‐5p is the critical effector molecule mediating the observed benefits.

The impact of miR‐211‐5p‐enriched exosomes on astrocyte activation was also significant. Elevated GFAP expression is a marker of reactive gliosis, which can exacerbate inflammation and impede neural recovery [21]. Treatment with modified exosomes led to decreased GFAP expression at both the mRNA and protein levels in the ipsilateral cortex. This finding highlights a regulatory role of miR‐211‐5p in modulating astrocyte reactivity, a feature not previously attributed to this miRNA in the context of TBI. Moreover, behavioral improvements in the Morris Water Maze align with these molecular changes, supporting a link between neuroinflammation, BBB dysfunction, and impaired cognition.

Clinically, these findings point toward a novel cell‐free therapeutic strategy for TBI. Current interventions remain largely supportive, and pharmacological agents targeting neuroinflammation or BBB integrity have yielded inconsistent results in human trials, in part due to poor specificity and delivery challenges [22]. The use of engineered exosomes as delivery vehicles for miRNAs offers a promising alternative, combining the ability to cross the BBB with endogenous biocompatibility and target cell interaction [23]. Importantly, miR‐211‐5p‐loaded exosomes can be produced from autologous or allogeneic glial cells, offering translational flexibility.

Exosome‐based therapies offer several translational advantages: They can be produced from autologous or allogeneic cell sources, engineered to carry specific therapeutic cargo, and administered systemically to reach injured brain regions. However, several translational considerations must be addressed before clinical application. First, scalable production methods for clinical‐grade exosomes need to be established, with standardized protocols for isolation, purification, and quality control. Second, while the dose used in this study (1 × 10^11^ particles) was based on a previous study [24], dose–response optimization and pharmacokinetic profiling will be necessary for human translation. Third, safety considerations related to AAV transduction of astrocytes must be carefully evaluated; alternative approaches such as transfection with synthetic miRNA mimics or liposome‐based packaging may offer safer production methods. Fourth, potential off‐target effects of systemically administered exosomes require comprehensive assessment, including biodistribution studies in non‐target organs and long‐term safety monitoring. Despite these challenges, the robust neuroprotective effects observed in this study warrant further preclinical development and eventual translation to clinical trials.

This study also addresses a critical knowledge gap regarding the molecular mechanisms by which astrocytes influence the post‐traumatic microenvironment. While reactive gliosis has been widely studied, few reports have dissected the role of astrocyte‐secreted miRNA cargo in modulating TBI pathology. Our data suggest that miR‐211‐5p not only alters astrocytic behavior but also regulates downstream pathways in endothelial cells and possibly microglia, thus exerting widespread effects on the injured brain milieu.

Nevertheless, limitations exist. The observation window of this study was limited to 14 days post‐injury (acute to subacute phase). While this timeframe is critical for secondary injury processes, including peak brain edema, maximal BBB disruption, and intense neuroinflammation, we did not assess long‐term pathological progression and functional recovery beyond 14 days. Therefore, we cannot fully determine whether the protective effects of EXO^miR^ persist in ameliorating chronic pathological changes following TBI. Future studies with extended follow‐up periods are needed to evaluate the durability of therapeutic benefits. We did not directly identify the downstream gene targets of miR‐211‐5p, and while its effects on protein and mRNA expression of MMP9 and AQP4 are evident, further mechanistic studies, including luciferase reporter assays and chromatin immunoprecipitation, may provide deeper insights into regulatory networks. Additionally, while in vivo fluorescence imaging demonstrated brain‐targeted enrichment of EXO^miR^ in injured regions, specific cellular uptake patterns (endothelial cells, astrocytes, microglia) were not delineated. Expanding these analyses in future work will strengthen the translational potential of this therapeutic approach. In addition, the long‐term effects of miR‐211‐5p on neurogenesis, synaptic plasticity, and functional integration remain to be studied. Optimization of dosing and timing regimens will also be essential before translation to clinical settings.

## 5 Conclusions

In conclusion, this study provides new evidence that targeted delivery of miR‐211‐5p via astrocyte‐derived exosomes can mitigate secondary injury processes following TBI. By demonstrating its capacity to suppress glial reactivity, preserve BBB integrity, reduce neuroinflammation, and improve cognitive outcomes, our findings support miR‐211‐5p as a promising therapeutic target. This work adds mechanistic depth to the understanding of astrocyte‐exosome signaling in CNS injury and opens avenues for developing precision exosome‐based therapies for neurotrauma.

## Author Contributions

Yan Chen, Xuan Yin, Erwei Zhang, Bin Li, Huijie Yu, Jiawang Qi, Minghao Liu, Meng Wang, Zhenzeng Fan, and Lijun Yang: Data curation, analysis, manuscript preparation. Zhenzeng Fan and Lijun Yang: Supervision.

## Funding

This work was supported by the Beijing – Tianjin – Hebei Cooperation Project (H2024206573, 24JCZXJC00200) and the 2023 Medical Science Research Project Plan of Hebei Province (20230657).

## Ethics Statement

Animal studies were approved by the Research Ethics Committee of the Second Hospital of Hebei Medical University (#2022‐R258). This study was performed in strict accordance with the NIH guidelines for the care and use of laboratory animals (NIH Publication No. 85–23 Rev. 1985).

## Consent

The authors have nothing to report.

## Conflicts of Interest

The authors declare no conflicts of interest.

## Supporting information


**Figure S1:** In vivo fluorescence imaging showing dynamic brain‐targeted enrichment of EXO^miR^ in TBI rats. EXOmiR was labeled with a near‐infrared fluorescent dye (DiR) prior to injection via the right jugular vein in TBI rats. In vivo fluorescence imaging was performed at the indicated time points to track the spatial–temporal distribution of EXO^miR^.
**Figure S2:** miR‐211‐5p inhibition attenuated EXOmiR‐mediated protection of blood–brain barrier (BBB) integrity as assessed by Evans blue assay. (A) Representative images of Evans blue extravasation in the brain tissues of TBI rats. Evans blue dye (2% in PBS) was intravenously injected 24 h before sacrifice to evaluate BBB permeability. (B) Quantitative analysis of Evans blue concentration in the ipsilateral cortex (μg/g tissue weight). N = 3 for each group. The date was shown with mean ± SD. *p < 0.05 from Brown–Forsythe ANOVA test followed by Dunnett's T3 multiple comparisons test.
**Figure S3:** miR‐211‐5p inhibition attenuated the suppressive effects of EXOmiR on the MMP9/AQP4 axis and neuroinflammation in TBI rats. (A–D) RT‐qPCR was used to analyze the expressions of miR‐211‐5p (A), mRNA expressions of MMP9 (B), AQP4 (C), and GFAP (D) in the ipsilateral cortex 3 days post‐TBI. Protein levels of pro‐inflammatory cytokines IL‐6 (E) and IL‐1β (F) in the ipsilateral cortex were quantified by ELISA. N = 3 for each group. The date was shown with mean ± SD. *p < 0.05 and **p < 0.01 from Brown–Forsythe ANOVA test followed by Dunnett's T3 multiple comparisons test.

## Data Availability

The data that support the findings of this study are available from the corresponding author upon reasonable request.
