# Supplementary material for: Astrocyte‐Derived Exosomal miR‐211‐5p Alleviates Blood–Brain Barrier Injury in a Rat Model of Traumatic Brain Injury
Source: CNS Neurosci Ther. 2026 Apr 9;32(4):e70858. doi: 10.1002/cns.70858 (PMC13064413; doi:10.1002/cns.70858)
Supplement: Supplementary file 1 — Figure S1: In vivo fluorescence imaging showing dynamic brain‐targeted enrichment of EXOmiR in TBI rats. EXOmiR was labeled with a near‐infrared fluorescent dye (DiR) prior to injection via the right jugular vein in TBI rats. In vivo fluorescence imaging was performed at the indicated time points to track the spatial–temporal distribution of EXOmiR. Figure S2: miR‐211‐5p inhibition attenuated EXOmiR‐mediated protection of blood–brain barrier (BBB) integrity as assessed by Evans blue assay. (A) Representative images of Evans blue extravasation in the brain tissues of TBI rats. Evans blue dye (2% in PBS) was intravenously injected 24 h before sacrifice to evaluate BBB permeability. (B) Quantitative analysis of Evans blue concentration in the ipsilateral cortex (μg/g tissue weight). N = 3 for each group. The date was shown with mean ± SD. *p < 0.05 from Brown–Forsythe ANOVA test followed by Dunnett's T3 multiple comparisons test. Figure S3: miR‐211‐5p inhibition attenuated the suppressive effects of EXOmiR on the MMP9/AQP4 axis and neuroinflammation in TBI rats. (A–D) RT‐qPCR was used to analyze the expressions of miR‐211‐5p (A), mRNA expressions of MMP9 (B), AQP4 (C), and GFAP (D) in the ipsilateral cortex 3 days post‐TBI. Protein levels of pro‐inflammatory cytokines IL‐6 (E) and IL‐1β (F) in the ipsilateral cortex were quantified by ELISA. N = 3 for each group. The date was shown with mean ± SD. *p < 0.05 and **p < 0.01 from Brown–Forsythe ANOVA test followed by Dunnett's T3 multiple comparisons test. [file CNS-32-e70858-s001.docx]

Supplementary materials


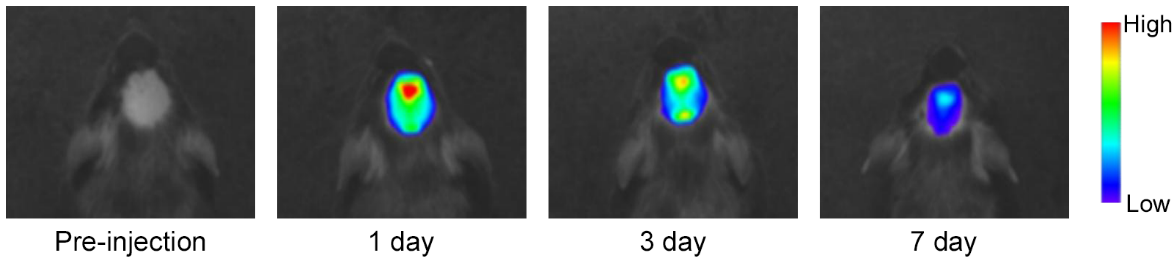
Figure S1. In vivo fluorescence imaging showing dynamic brain-targeted enrichment of EXO^miR^ in TBI rats. EXOmiR was labeled with a near-infrared fluorescent dye (DiR) prior injection via the right jugular vein in TBI rats. In vivo fluorescence imaging was performed at the indicated time points to track the spatial-temporal distribution of EXO^miR^.


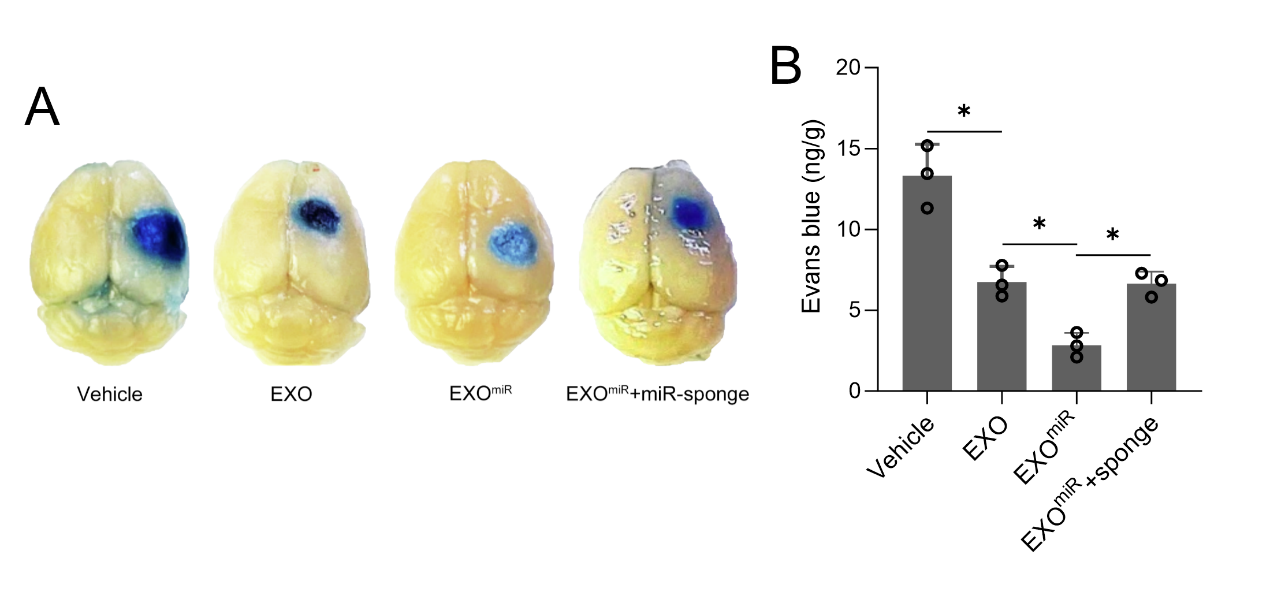
Figure S2. miR-211-5p inhibition attenuated EXO^miR^-mediated protection of blood-brain barrier (BBB) integrity as assessed by Evans blue assay. A. Representative images of Evans blue extravasation in brain tissues of TBI rats. Evans blue dye (2% in PBS) was intravenously injected 24 h before sacrifice to evaluate BBB permeability. B. Quantitative analysis of Evans blue concentration in the ipsilateral cortex (μg/g tissue weight). N =3 for each group. Date was shown with mean ± SD. *p < 0.05 from Brown-Forsythe ANOVA test followed with Dunnett's T3 multiple comparisons test.


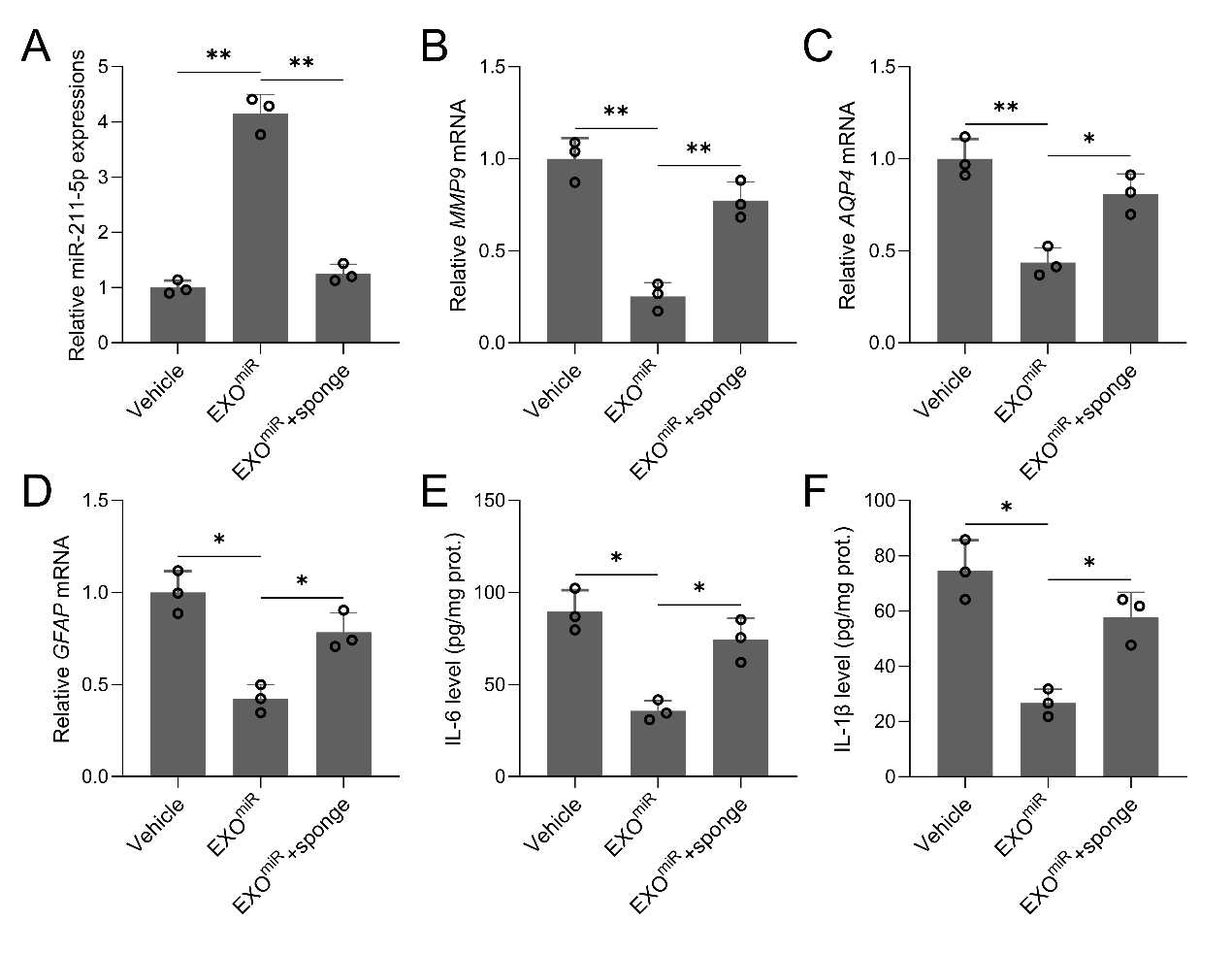
Figure S3. miR-211-5p inhibition attenuated the suppressive effects of EXO^miR^ on MMP9/AQP4 axis and neuroinflammation in TBI rats. A-D. RT-qPCR was used to analyze the expressions of miR-211-5p (A), mRNA expressions of MMP9 (B), AQP4 (C) and GFAP (D) in the ipsilateral cortex 3 days post-TBI. Protein levels of pro-inflammatory cytokines IL-6 (E), IL-1β (F) in the ipsilateral cortex were quantified by ELISA. N =3 for each group. Date was shown with mean ± SD. *p < 0.05 and **p < 0.01 from Brown-Forsythe ANOVA test followed with Dunnett's T3 multiple comparisons test.
